# Kinetic, Isotherm, and Thermodynamic Studies for Ag(I) Adsorption Using Carboxymethyl Functionalized Poly(glycidyl methacrylate)

**DOI:** 10.3390/polym10101090

**Published:** 2018-10-01

**Authors:** Jiling Zhao, Shixing Wang, Libo Zhang, Chen Wang, Bing Zhang

**Affiliations:** 1 State Key Laboratory of Complex Nonferrous Metal Resources Clean Utilization, Kunming University of Science and Technology, Kunming 650093, China; 15687884157@163.com; 2 National Local Joint Laboratory of Engineering Application of Microwave Energy and Equipment Technology, Kunming 650093, China; wc932608335@163.com (C.W.); 15198750002@163.com (B.Z.); 3 Faculty of Metallurgical and Energy Engineering, Kunming University of Science and Technology, Kunming 650093, China; 4 Key Laboratory of Unconventional Metallurgical Ministry of Education, Kunming 650093, China

**Keywords:** polymer, Ag (I), removal, mechanism, adsorption

## Abstract

Industrial wastewater contains large amounts of silver ions. Here, a new adsorbent was synthesized by functionalizing poly(glycidyl methacrylate) with carboxymethyl groups. The adsorbent was used to recover Ag(I) in wastewater. Fourier transform infrared spectroscopy, zeta potential, scanning electron microscopy, and X-ray photoelectron spectroscopy were used to characterize the adsorbent. The experimental parameters affecting the adsorption are solution pH, contact time, and initial silver ion concentration. The optimum pH for adsorption of Ag(I) is pH 4. The maximum adsorption capacity at pH 4 is 157.05 mg/g, and the adsorption reaches equilibrium at 300 min. The kinetics and isotherms of the adsorption process were described by pseudo second-order, Langmuir and D-R models, respectively. The adsorption process was a single layer chemical adsorption, exothermic, feasible, and spontaneous. The adsorption mechanism is electrostatic or chelation. The adsorbent selectively absorbed Ag(I) from coexisting ions (Cu^2+^, Ni^2+^, Co^2+^, Zn^2+^). Finally, the removal rate of silver ions decreased from 79.29% to 65.01% after four repetitive experiments, which proved that the adsorbent had good reusability. The adsorbent has great potential benefit in removing Ag(I).

## 1. Introduction

In many parts of the world, clean and safe water is becoming scarcer, mainly due to pollution [1,2]. Wastewater discharged in many industries, such as metallurgy, mineral processing, tanning, chemical manufacturing, and battery manufacturing, contains one or more toxic metal ions [3]. Among them, silver is one of the most toxic metals due to its instability [4]. If the wastewater is not treated in time, Ag(I) will circulate with the water, immerse into the groundwater or be exposed to the air, and may result in causing various diseases. [5,6,7,8]. On the other hand, due to its excellent properties, silver is widely used in medical products, photography, electronics, and other important fields [9,10]. Therefore, the recovery of silver from wastewater has high economic and environmental significance.

The developed treatment technologies for silver from wastewater include ion exchange, solvent extraction, precipitation, electrolysis, and adsorption [11,12,13,14]. Among these technologies, adsorption is considered a promising method for removing Ag(I) because of its high absorption capacity, reusability, low cost, and environmental friendliness [15,16,17]. Currently, various adsorbents have been used such as silica gel, activated carbon, fiber, biomaterials, and polymeric materials [18,19,20,21]. In contrast, polymer resins with various functional groups have high selectivity, easy desorption, and good structural diversity [20]. Recently, poly(glycidyl methacrylate) (PGMA)-based chelating resins have received a lot of attention and were successfully applied to the adsorption of heavy metals, noble metals, and dyes in wastewater [22,23,24] because PGMA-based chelating resins have acid and alkaline resistance, good mechanical strength, high tensile strength, wear resistance, and low cost [25]. The most outstanding characteristics of PGMA is that it contains highly reactive epoxy groups, has a porous structure, and is easily functionalized with various group by a single chemical reaction [26,27]. Therefore, PGMA-based chelating adsorbents have good industrial application prospects in the removal of metal ions from wastewater.

In recent years, PGMA has been functionalized by coordination groups containing donor atoms such as O, N, S and P for removal of different metal ions. Liu et al. removed Cu(II) by poly(glycidyl methacrylate) (PGMA) modified with diethylenetriamine [28]. Krajnc et al. functionalized PGMA with penta-erythritol tetrakis(3-mercaptopropionate), 1,9-nonanedithiol, and 2-aminobenzenethiol for removal of silver, lead, and cadmium from water [29]. Polyethylenimine-grafted poly(glycidyl methacrylate) (PGMA-PEI) microspheres adsorbed selectively Cr(VI) from coexisting ions of K^+^, Na^+^, Ca^2+^, Cu^2+^, Cl^−^, NO_3_^−^, H_2_PO_4_^−^, and HPO_4_^2−^ [30]. A triethylene teramine (TETA) functionalized magnetic poly (glycidyl methacrylate) (PGMA) nano-absorbent showed high selective adsorption for Hg(II) and good regeneration performance [31]. In brief, surprisingly high selectivity and efficiency of PGMA-based chelating adsorbents could be obtained by grafting specific functional groups.

In this work, we developed a new adsorbent by functionalizing PGMA with carboxymethyl to selectively absorb Ag(I) from wastewater. The adsorbent was characterized by X-ray photoelectron spectroscopy (XPS), Fourier transform infrared spectroscopy (FT-IR), zeta potential, and scanning electron microscopy (SEM). The effects of pH, initial Ag(I) concentration, and reaction time were investigated. At the same time, the properties of the adsorbent were investigated by selectivity, reusability, thermodynamic, isotherms, and kinetics. The adsorption mechanisms were also studied.

## 2. Materials and Methods

### 2.1. Materials

Glycidyl methacrylate (GMA), azobisisobutyronitrile (AIBN), polyethyleneimine, bromoacetic acid (98%), anhydrous ethanol (99%) and polyvinyl pyrrolidone (PVP) were purchase from Aladdin Chemistry Co. Ltd., Shanghai, China. Sodium carbonate anhydrous, potassium carbonate, and carbon disulfide were obtained from Tianjin Chemical Reagent Co. Ltd., Tianjin, China. Silver Nitrate was purchased from Aladdin Chemistry Co. Ltd., Shanghai, China. All of the chemicals were analytical reagent. The pH value of solutions was adjusted by HNO_3_ and NaOH solutions.

### 2.2. The Synthesis Process of Carboxymehyl Functionalized PGMA

**Scheme 1:**
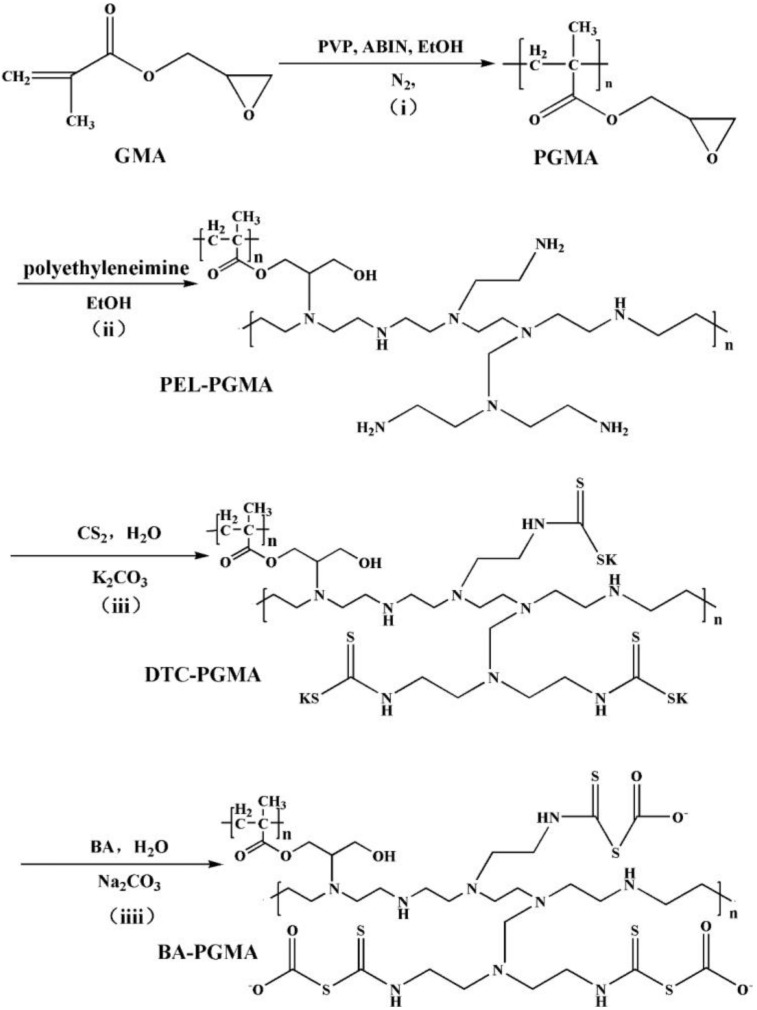
The synthesis route of BA-PGMA (poly(glycidyl methacrylate) (PGMA) with carboxymethyl groups).

Scheme 1 presents the functionalization process of PGMA with carboxymethyl groups (BA-PGMA). Firstly, GMA (10.0 g), PVP (3.0 g), and AIBN (0.12 g) were added to 150 mL of ethanol solution and the reaction was carried out at 70 °C for 5 h under N_2_ with stirring. After washing with ethanol for several times, the solid product (PGMA) was dried under vacuum at 50 °C for 24 h. Second, PGMA (3 g), polyethyleneimine (6 g) and ethanol (100 mL) were added to a three-necked flask. The mixture was stirred for 48 h at 70 °C. Then, the suspension was centrifuged (9000 rpm, 15 min) and the precipitate was washed with ethanol and water until the water was neutral and then dried at 60 °C for 12 h. The obtained product was referred to as PEL-PGMA. Then, PEL-PGMA (2.5 g), carbon disulfide (1.0 g), and potassium carbonate (2.0 g) were added to a three-necked flask containing 40 mL of deionized water. The mixture was stirred at room temperature for 12 h. Then, the precipitate was washed with deionized water until the water was neutral and dried at 60 °C for 12 h. The product was defined as DTC-PGMA. Lastly, DTC-PGMA (3.2 g), deionized water (40 mL), sodium carbonate (4.0 g), and bromoacetic acid (4.0 g) were added into a three-necked flask. The mixture was stirred at 35 °C for 12 h. The suspension was then centrifuged and the precipitate was washed with deionized water until the filtrate was neutral. The final product was defined as BA-PGMA.

### 2.3. Characterization

The chemical forms of the functional groups were analyzed using FT-IR (Nicolet iS50, Thermo Scientific Co., Waltham, MA, USA) and XPS (ESCALab220i-XL, Thermo Scientific Co., Waltham, MA, USA). The microstructure of the sample was characterized by SEM and energy dispersive spectroscopy (EDS) (FEI/Philips XL30, Phenom ProX, Royal Dutch Philips Electronics Ltd., Amsterdam, The Netherlands). Zeta potential of the adsorbent was measured by a high sensitivity Zeta potential analyzer (Brookhaven Instruments Co., Austin, TX, USA). The concentrations of metal ions were detected by an inductively coupled plasma optical emission spectrometer (ICP) (Leeman Prodigy 7, Teledyne Leeman Labs., Hudson, NH, USA).

### 2.4. Adsorption Experiment

The experimental parameters included pH, adsorption time, and initial Ag(I) concentration. An amount of 10 mg of BA-PGMA was added to 15 mL of Ag(I) solution. The experiment was performed in a thermostatic steam bath shaker at an oscillating speed of 300 rpm and at room temperature. The supernatant was separated and the concentration of Ag(I) in the solution was determined using ICP. The removal rate (*R*) of Ag(I) was calculated by Equation (1):

(1) $$R=\frac{C_{\alpha }-C_{\beta }}{C_{\alpha }}\times 100\%$$

The adsorption capacity (*U*) of Ag(I) on BA-PGMA at equilibrium was calculated by Equation (2):

(2) $$U=\frac{C_{\alpha }-C_{\beta }}{m}\times V$$

Here, *C*_α_ (mg/L) represented the initial concentration of Ag(I), *C*_β_ (mg/L) represented the concentration of Ag(I) after adsorption, *U* represented the adsorption capacity. *V* (L) represented the volume and m (g) represented the mass of BA-PGMA.

## 3. Results and Discussion

### 3.1. Characterization of BA-PGMA

**Figure 1:**
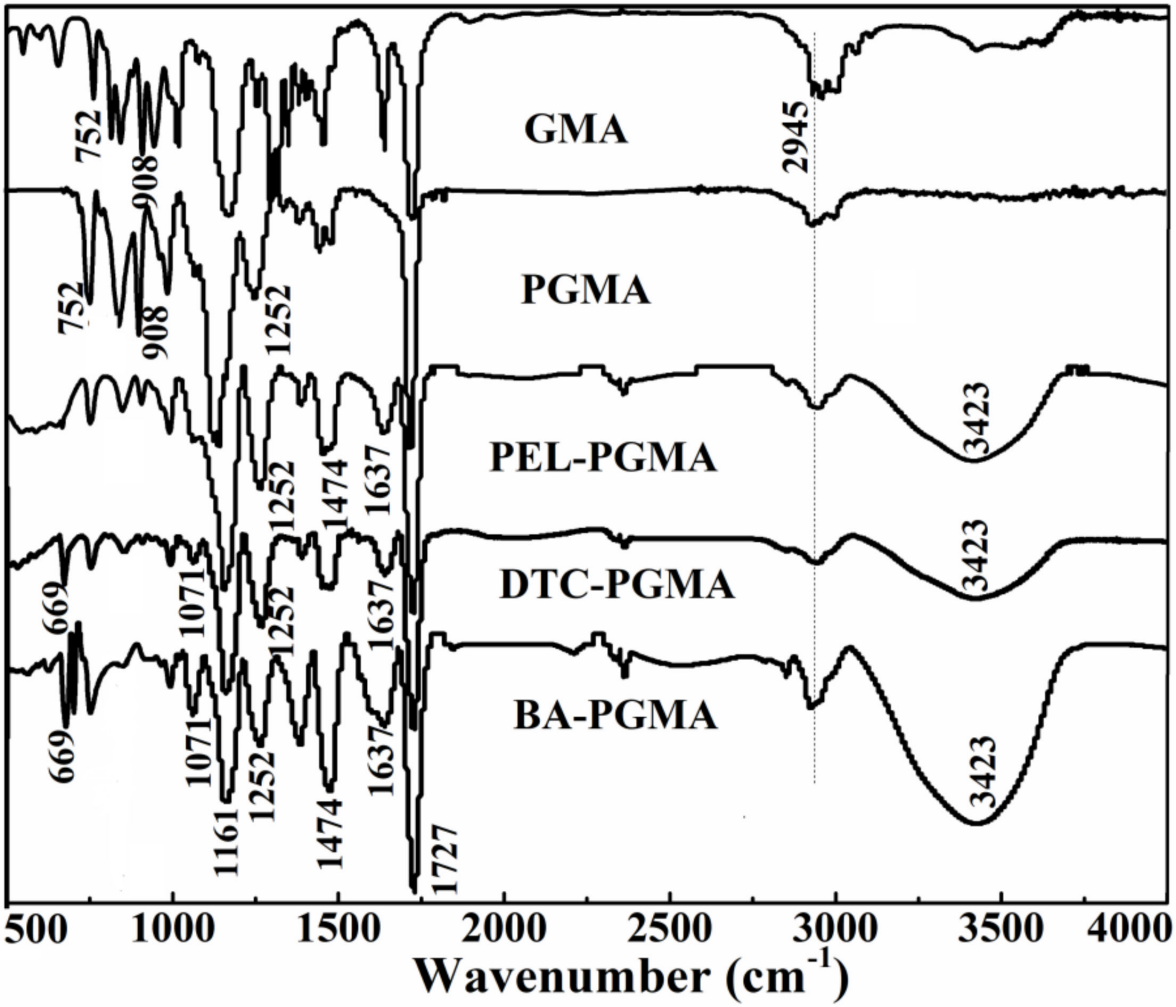
Fourier transform infrared spectroscopy (FT-IR) spectra of GMA, PGMA, PEL-PGMA, DTC-PGMA and BA-PGMA.

The FT-IR spectra of GMA, PGMA, PEL-PGMA, DTC-PGMA, and BA-PGMA are showed in Figure 1. The peaks at 751 cm^−1^, 1161 cm^−1^, 1252 cm^−1^, 1727 cm^−1^ and 2945 cm^−1^ were attributed to the stretching vibrations of C–O–C, C–O, C–C, C=O and C-H, respectively [32]. The peak of 908 cm^−1^ was due to the epoxy group of GMA and PGMA. After reacting with polyethyleneimine, the epoxy group was opened, resulting in the disappearance of the epoxy peak. In the infrared spectrum of PEL-PGMA, DTCPGMA, and BA-PGMA, the new peak at 1474 cm^−1^ was due to the stretching vibration of C–N. The vibration belts at 3430 cm^−1^ and 1637 cm^−1^ were t O–H deformation vibration. The new peaks at 669 cm^−1^ and 1071 cm^−1^ were due to the stretching vibration of C–S and C=S of DTC-PGMA and BA-PGMA. For BA-PGMA, the O–H deformation vibrating at 3430 cm^−1^ and 1637 cm^−1^ was significantly enhanced due to the C–O and C=O bonds in acetic acid. The results indicated that BA-PGMA had been successfully synthesized.

**Figure 2:**
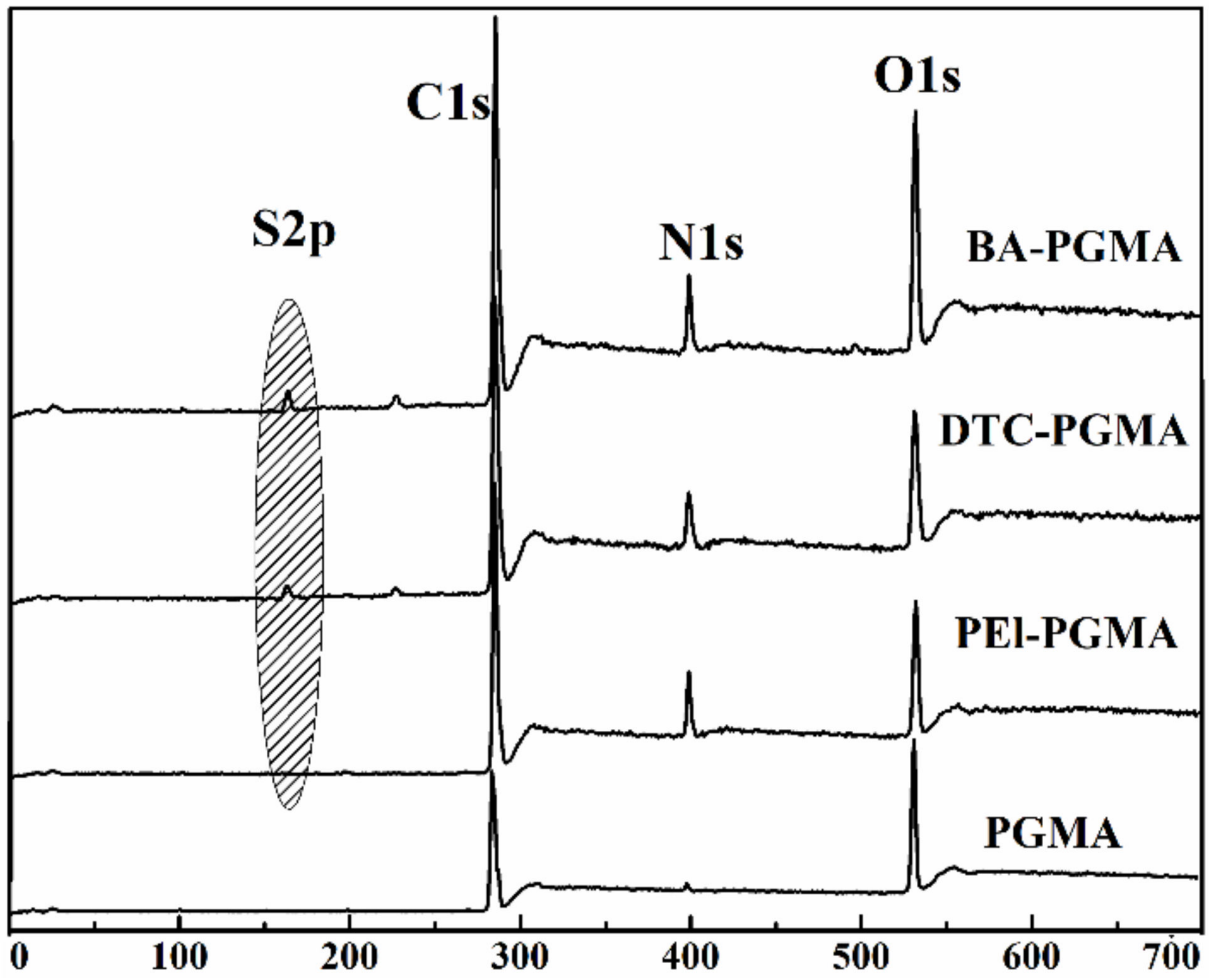
X-ray photoelectron spectroscopy (XPS) survey scan of PGMA, PEL-PGMA, DTC-PGMA, and BA-PGMA.

**Figure 3:**
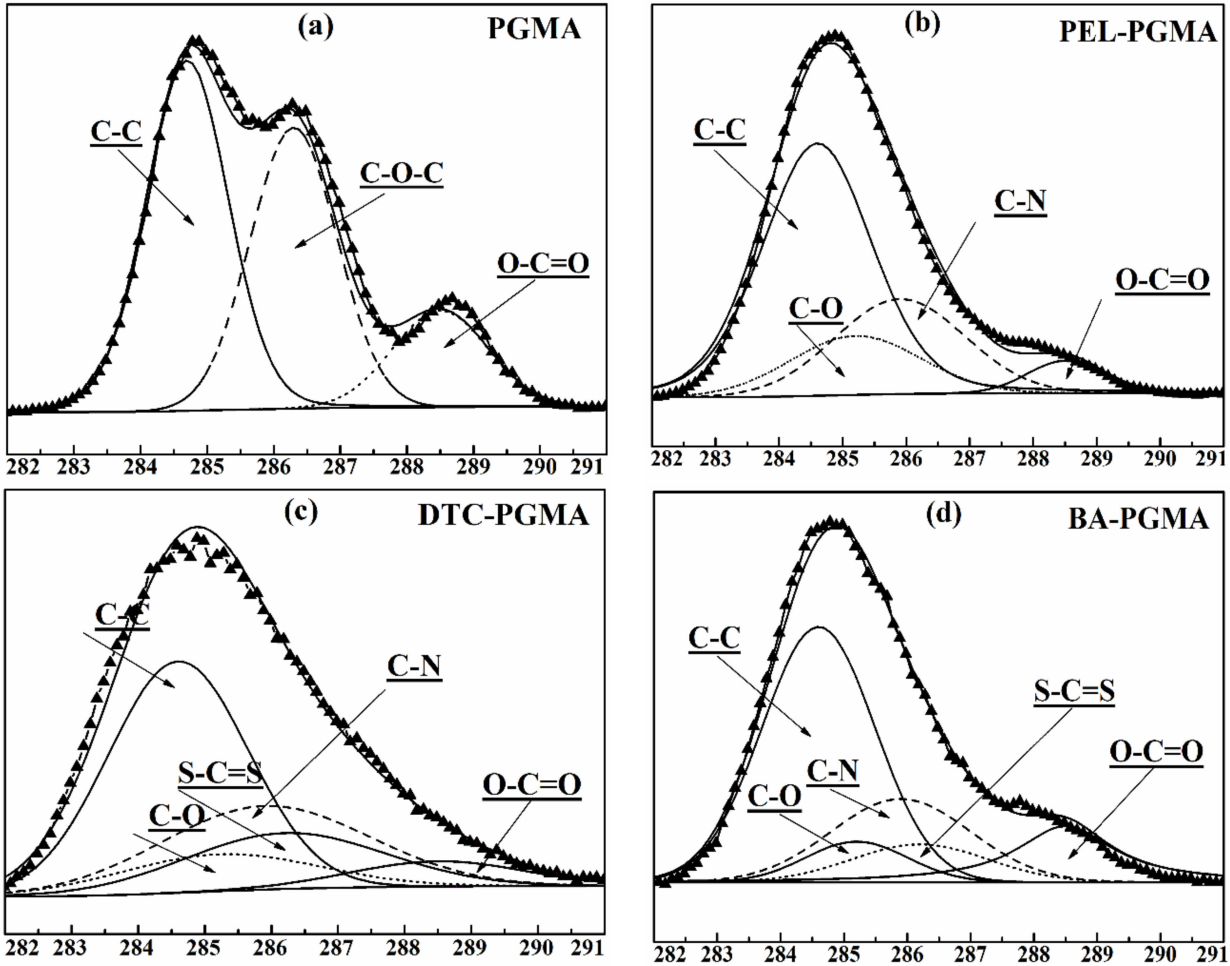
C1s spectra of PGMA (**a**), PEL-PGMA (**b**), DTC-PGMA (**c**), BA-PGMA (**d**).

X-ray photon spectroscopy was used to determine the chemical composition of the material. Figure 2 shows the peaks of N1s and S1s in the modified material. Figure 3 shows the C1s spectra. The C1s of PGMA presents the C–C (286.2 eV), O–C=O (288.5 eV) and C–O–C (285.2eV) groups (Figure 3a) [33]. After polyethyleneimine modification, a new C-N (285.9 eV) peak appeared in PEL-PGMA (Figure 3b) [34]. Comparing with PEL-PGMA, DTC-PGMA shows the S–C=S bond (286.2 eV) (Figure 3c) [35]. In BA-PGMA, the area of the O–C=O group was larger than that of DTC-PGMA because the number of O–C=O groups increased (Figure 3d).

### 3.2. Effect of Experimental Parameters on Adsorption of Ag(I)

#### 3.2.1. Effect of pH

**Figure 4:**
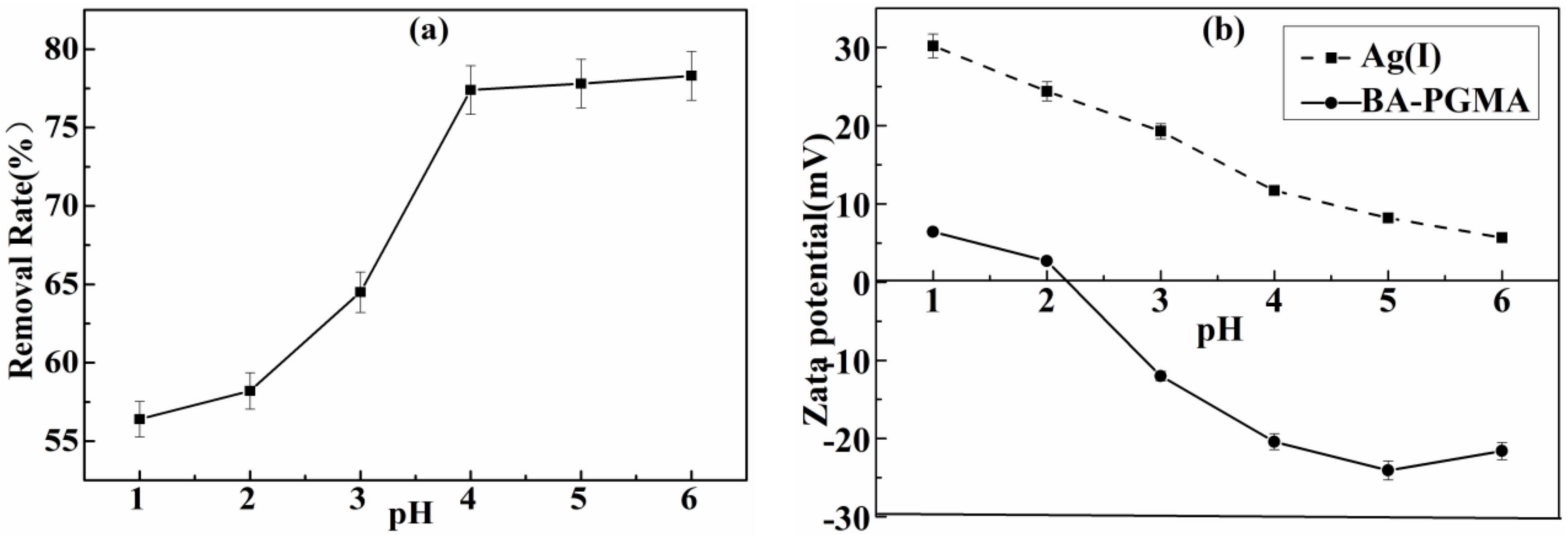
Effect of pH on the removal rate of Ag(I) (**a**) and zeta potential of BA-PGMA (**b**).

As one of the main factors affecting the adsorption of Ag(I) by BA-PGMA, the pH of the solution can promote the dispersion of the functional groups of the adsorbent and adjust the resulting electrostatic adsorption or complexation reaction. We studied the effect of solution pH values from 1 to 6 on the removal rate of Ag(I). The hydrolysis of Ag(I) occurs in the solution when the pH is greater than 6. An amount of 10 mg of BA-PGMA was added to 15 mL of silver solution (100 mg/L), and oscillated for eight hours at room temperature. Then, the solution was centrifuged to determine the concentration of Ag(I). As shown in Figure 4a the removal rate of Ag^+^ was relatively low when the pH was in the range of 1 to 2, and as the pH increased, the removal rate of Ag was improved and reached maximum (76.4%) at pH 4. So, we chose pH 4 as the optimal pH in the following experiments. In order to verify the adsorption mechanism, Zeta potential was used to study the charge type of the BA-PGMA surface (Figure 4b). The isoelectric point of BA-PGMA was approximately 2.1, which revealed that the BA-PGMA adsorbent was positively charged at pH < 2.1 and negatively charged at pH > 2.1. Most of the amine groups on the adsorbent surface were protonated at pH < 2.1, which induced an electrostatic repulsion to the silver ions. There was no electrostatic attraction between BA-PGMA and Ag(I) at this time. The deprotonation of amine groups induced the electrostatic attraction toward the Ag^+^ at pH > 2.1. In addition, at low pH, the higher concentration of H^+^ ions will compete with Ag^+^ for the complex sites, resulting in the decrease of adsorption capacity of the silver ions [36]. In order to study the stability of BA-PGMA in acidic solution, BA-PGMA was soaked in an aqueous solution at pH 4 for 24 h. After filtering and drying, the soaked BA-PGMA was used to adsorb silver ions. The removal rate of silver ion by the soaked BA-PGMA was 71.7%, which was close to that of BA-PGMA. Therefore, BA-PGMA is stable in acidic solution.

#### 3.2.2. Effect of Time on Adsorption Capacity of Ag(I) and Kinetics

**Figure 5:**
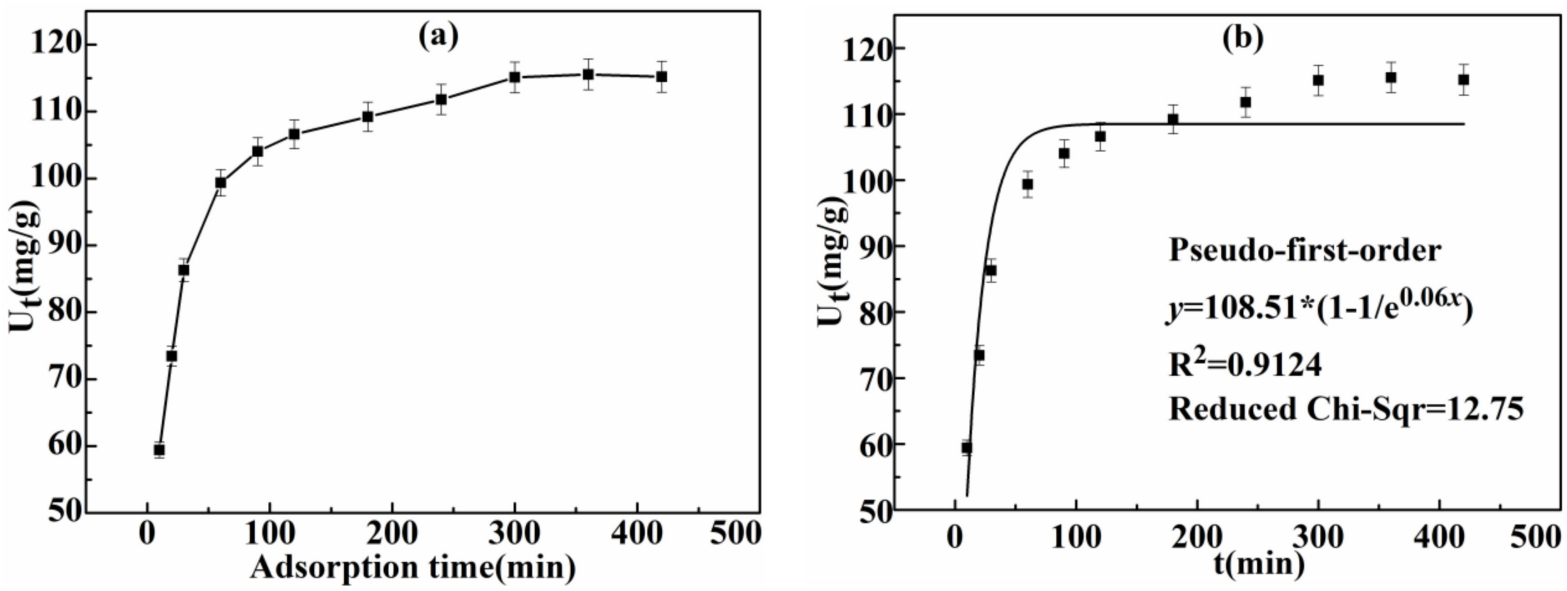

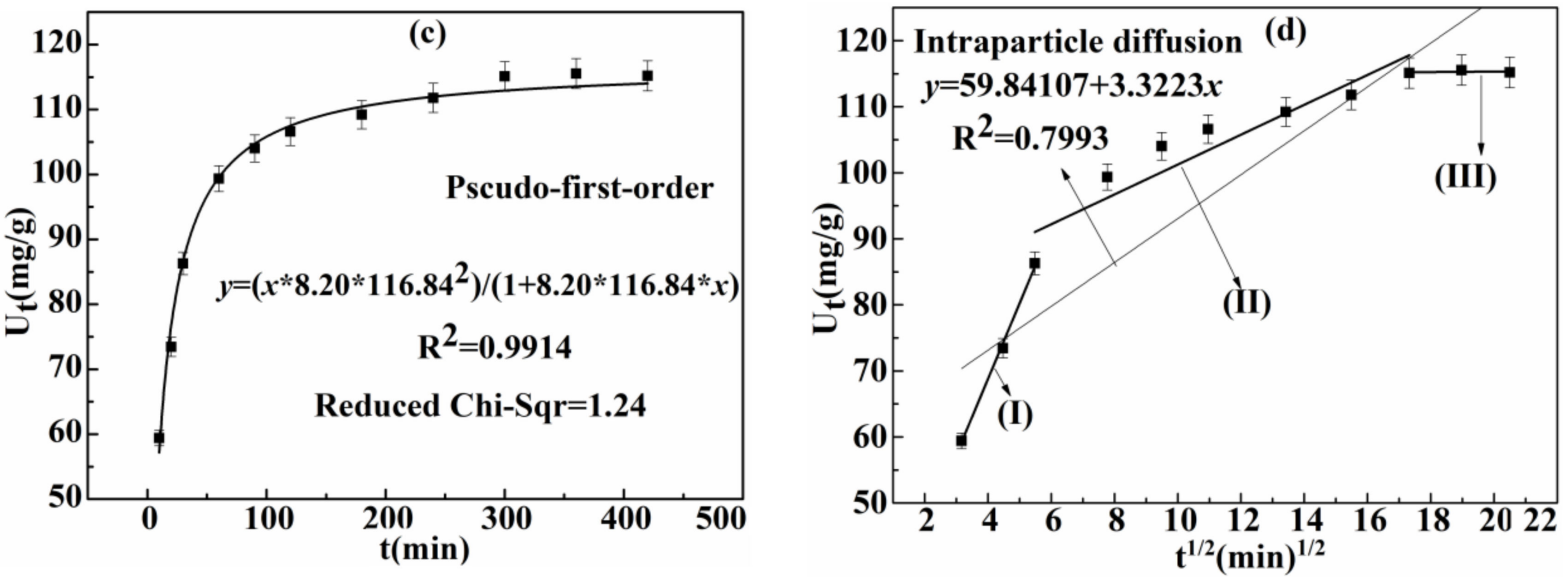
Effect of adsorption time on adsorption of Ag(I) by BA-PGMA (**a**) and kinetic model (pseudo-level (**b**), pseudo-secondary (**c**), intra-particle diffusion (**d**)).

Figure 5a shows the effect of adsorption time on the adsorption capacity of silver ions when the initial concentration is 100 mg/L at pH 4. In the first ten minutes, the adsorption rate of Ag(I) was very fast because there were enough active sites on BA-PGM. After ten minutes, the adsorption amount of Ag(I) gradually increased with time. The adsorption capacity increased slightly with the passage of time, and the adsorption reached equilibrium at 300 min.

We used the pseudo first-order, pseudo second-order and intraparticle diffusion kinetics model to study the adsorption mechanism. The pseudo-first-order model is mainly physical adsorption. The pseudo-first-order model assumed the occupancy of adsorption sites was directly proportional to the driving forces and the number of unoccupied sites, respectively [37]. The adsorption process explained by the pseudo second-order model was mainly chemical adsorption [38]. In order to determine whether the adsorption process of Ag(I) was related to the rate, the mechanism of intraparticle diffusion model was used [39]. The Equations (3)–(5) were the pseudo-first-order, pseudo-second-order, and intraparticle diffusion kinetic models, respectively:

(3) $$U_{t}=U_{e}\left(1-\frac{1}{e^{K_{a}t}}\right)$$

(4) $$U_{t}=\frac{t\cdot K_{b}\cdot U_{e}{}^{2}}{1+K_{b}\cdot U_{e}\cdot t}$$

(5) $$U_{t}=K_{c}t^{1/2}+C$$

where, *U*_e_ (mg/g) was the equilibrium adsorption capacity; *U_t_* (mg/g) was the uptake capacity at time *t* (min). *K*_a_ (g/(mg·min)), and *K*_b_ (g/mg·min) were the adsorption rate constants of pseudo-primary and pseudo-secondary models. *K_c_* (mg/g·min^1/2^) was the intraparticle diffusion rate constant, and *C* was relative to the liquid film thickness index.

The obtained experimental data were respectively fitted using three kinetic models (Figure 5b–d). The kinetic parameters for the three models are listed in Table 1. In contrast, the kinetics of BA-PGMA adsorption of Ag(I) was more consistent with the pseudo second-order model, because the R^2^ value (0.9914) of the pseudo-quadratic model was higher than those of other models. In addition, the adsorption capacity of the second-order model was 116.84 mg/g, which was close to the experimental data (117.15 mg/g). These results demonstrate that the adsorption of Ag(I) conforms to the pseudo-secondary model, and the process was controlled by chemisorption. This means that the Ag(I) in the solution interacted with the functional groups on the BA-PGMA and they shared an electron pair with each other. In addition, the intraparticle diffusion model can be divided into three processes (Figure 5d and Table 2). In stage I (*t* ≤ 30 min), the adsorption capacity of Ag(I) increased rapidly because there were enough active sites on the surface of BA-PGMA. In the stage II (30 ≤ *t* ≤ 300 min), the adsorption capacity of silver ion was reduced compared to the first stage, because the active sites decreased as the adsorption time increased. In stage III (300 min ≤ *t* ≤ 420 min), the adsorption capacity was basically unchanged, and the adsorption equilibrium was reached. First, the fitting factor (*R*^2^) of the stage II was higher than the other two stages. At this stage, membrane diffusion (external mass transfer) dominates at the beginning. Then, after the active site of the BA-PGMA surface was saturated, Ag(I) migrates to the inner surface, and intraparticle diffusion controls the adsorption rate. In addition, the straight line fitted by these three stages does not pass through the origin. It can be concluded that the adsorption process was not a single process, and both surface adsorption and intraparticle diffusion processes affected the adsorption rate at different stages [40].

#### 3.2.3. Effect of Initial Concentration of Ag(I) and Adsorption Isotherm

**Figure 6:**
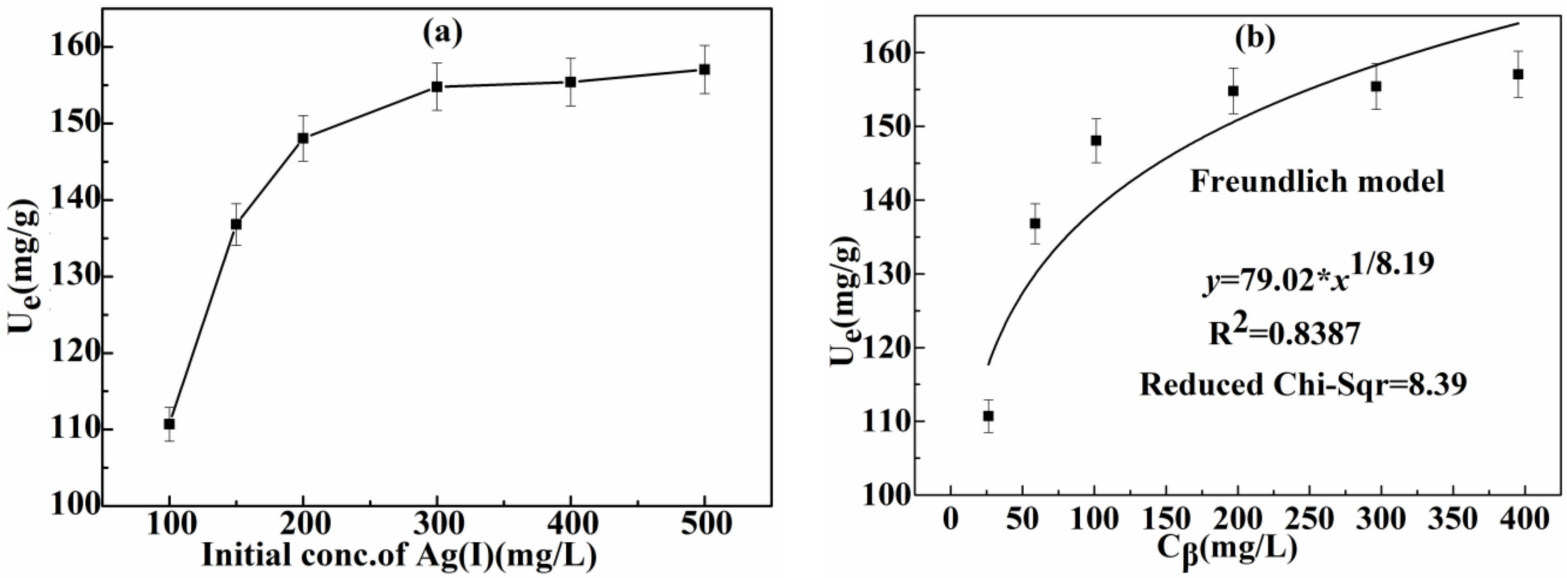

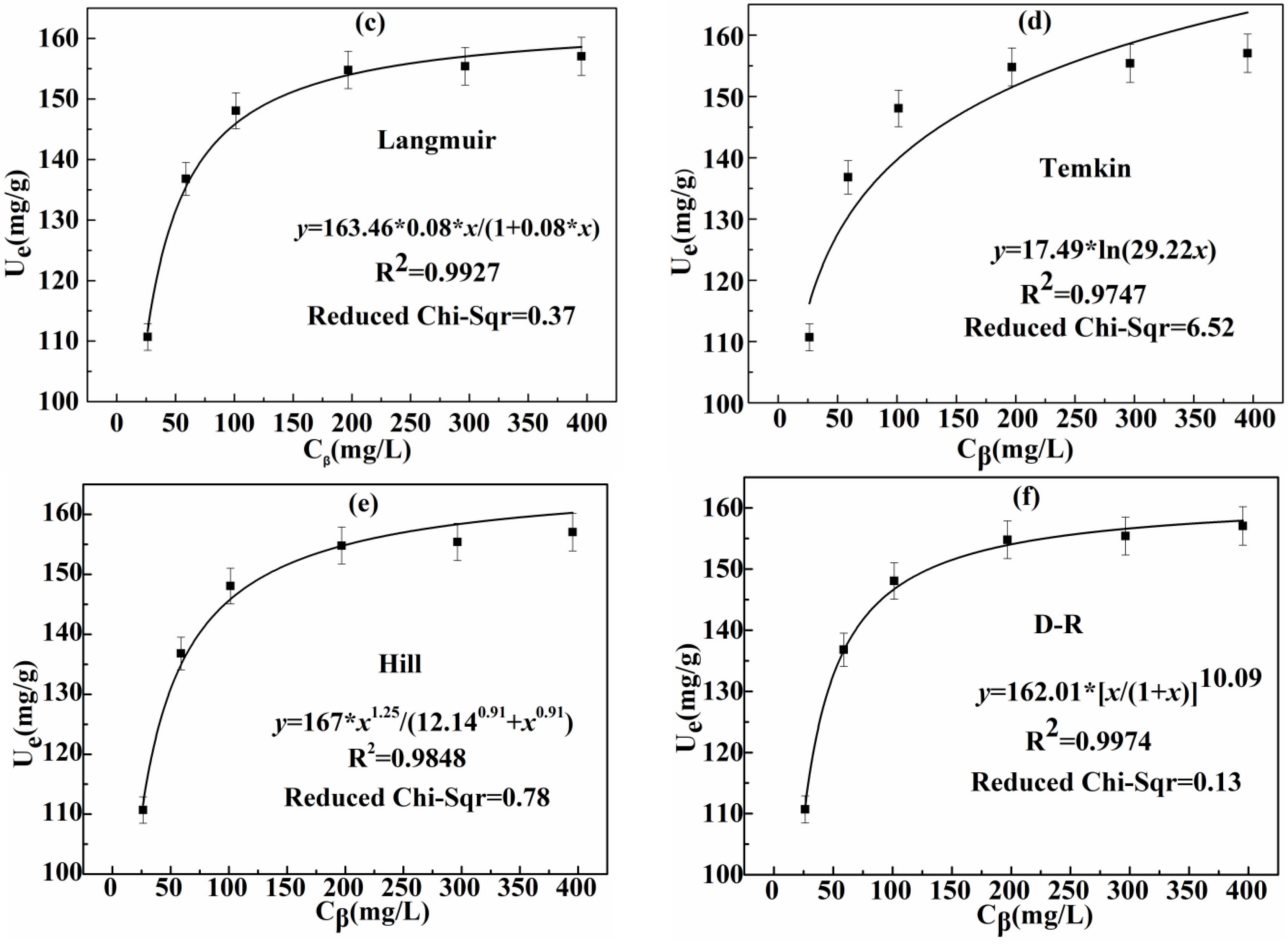
Effect of initial concentration of Ag(I) (**a**), adsorption isotherm, Freundlich (**b**), Langmuir (**c**), Temkin (**d**), Hill (**e**), and D-R (**f**).

In order to study the effect of the initial silver ion concentration on the adsorption, 10 mg of BA-PGMA was added to a series of 15 mL Ag(I) solutions with different concentrations at pH 4. As shown in Figure 6a, the absorption capacity of Ag(I) gradually increased with the increase of the silver ion concentration. At a concentration of 300 mg/L, adsorption equilibrium was reached. Above this value, there was no significant change in the adsorption capacity of Ag(I). When the Ag(I) concentration was less than 300 mg/L, there were enough active adsorption sites on the surface of BA-PGMA to adsorb Ag(I). When the Ag(I) concentration was more than 300 mg/L, the available adsorption sites of Ag(I) were saturated, and there was no active site for Ag(I) adsorption. The maximum uptake of silver was 157.05 mg/L, indicating that BA-PGMA was an effective adsorbent for Ag(I). It shows it has a great potential and application value in the recovery of Ag(I). 

Adsorption isotherms can indicate the interaction mechanism between BA-PGMA and Ag(I), as well as the change of adsorption capacity with the concentration of the adsorbed species. The adsorption capacity and equilibrium concentration of Ag(I) at 298 K and pH 4 were described using the Freundlich, Langmuir, Temkin, Hill, D-R isothermal models. The Freundlich isotherm model has a wide range of applications in certain areas. This model assumes that the adsorption is a multilayer sorption on heterogeneous surfaces and describes that the adsorption is non-ideal and reversible [41]. The linear mathematical expression is as shown in the Equation (6). *U*_e_, *C*_β_, and *K*_h_ represented the equilibrium adsorption capacity and concentration of Ag(I) after adsorption and the constant of this model. The slope 1/n is an indicator of adsorption intensity of surface heterogeneity [42].

(6) $$U_{e}=K_{h}\cdot C_{\beta }{}^{1/n_{1}}$$

The Langmuir isotherm model explained that the adsorption process was a monolayer adsorption on the homogeneous surfaces. Its equation was used to evaluate the adsorption capacities of the adsorbents [43]. The nonlinear expression of Langmuir is shown in Equation (7). Where *K_i_* was the constant of this model and *U*_m_ (mg/g) was the monolayer adsorption capacity of Ag(I). It was also the maximum adsorption capacity of single layer adsorption.

(7) $$U_{e}=\frac{U_{m}\cdot K_{i}\cdot C_{\beta }}{1+K_{i}\cdot C_{\beta }}$$

Adsorption heat linearly decreased from the reaction between the adsorbate and the adsorbent under Temkin isotherm conditions [44]. The mathematical expression of this model is Equation (8). *V_T_* and *K*_j_ are the constants of the Temkin model.

(8) $$U_{e}=V_{T}\cdot \ln\left(K_{j}\cdot C_{\beta }\right)$$

The Hill isothermal model assumes that one active site can absorb multiple ions, indicating a driving relationship between different adsorbates on the same uniform surface and the binding sites [45]. The nonlinear expression of the Hill mode is shown in Equation (9). Here, *K*_L_ and *N* are the constants of this model.

(9) $$U_{e}=\frac{U_{m}\cdot C_{\beta }{}^{N}}{1+K_{L}\cdot C_{\beta }{}^{N}}$$

The D-R model can be used to indicate the adsorption characteristics and the free energy of the adsorbate. It can also be used to analyze adsorption mechanisms on homogeneous or heterogeneous surfaces [46]. The nonlinear expression of the model is shown in Equation (10). Here, ω and *U***_m_** are the maximum adsorption capacity and constant, respectively. In addition, the free energy of adsorbed ions per molecule can be calculated by Equation (11). Free energy (*E*) was used to determine whether the adsorption process was physical or chemical adsorption. The value of 1–8 kJ/mol (*E*) explains that the adsorption is a physical process, 8–16 kJ/mol indicates that the adsorption is electrostatic interaction, and the chemical adsorption results in >16 kJ/mol.

(10) $$U_{e}=U_{m}\cdot {\left(\frac{C_{\beta }}{1+C_{\beta }}\right)}^{\omega RT}$$

(11) $$E=\frac{1}{\sqrt{2\omega }}$$

Figure 6 shows five models (Freundlich, Langmuir, Temkin, Hill, and D-R). Table 3 lists the relevant parameters for the five models. By comparing the five models, it can be found that the correlation coefficient values of Langmuir and D-R were relatively close (*R*^2^_L_ = 0.9927, *R*^2^_D_ = 0.9974) and greater than these of the other models. The maximum adsorption capacity (U_m_) calculated by the Langmuir model and the D-R model was 163.46 mg/g and 162.01 mg/g, which was close to the experimental data (157.05 mg/g). Therefore, the Langmuir and D-R models are in line with this study. It indicated that the active adsorption sites on BA-PGMA were homogeneous. At the same time, the value of free energy ω in the D-R model was 11.04 kJ/mol, indicating that the adsorption mechanism was electrostatic interaction or chelation.

To further investigate the adsorption process, the Langmuir isotherm model was used to estimate the value of the separation factor constant (*R*_L_) and to demonstrate the applicability of BA-PGMA to ions during adsorption. When *R*_L_ >1 it was not applicable, *R* = 1 it was linear, 0 < *R* < 1 it was applicable and *R* = 0 it was irreversible [47]. The mathematical expression for the coefficient *R*_L_ is as shown in Equation (12).

(12) $$R_{L}=\frac{1}{1+K_{s}\cdot C_{\alpha }}$$

Here, *C*_α_ is the initial concentration of silver ions and *K*_s_ is a constant. The *R*_L_ values for different initial Ag(I) concentrations are listed in Table 4, all of which are greater than zero and less than one. As the concentration of Ag(I) increases, the *R*_L_ value decreases gradually, indicating that the higher the concentration of Ag(I), the more favorable the adsorption.

Table 5 lists the maximum adsorption capacities reported for other adsorbents in the literature. It can be seen that the BA-PGMA adsorbent has a large adsorption capacity for Ag(I) and has great potential in the field of recycling and utilization of Ag(I).

#### 3.2.4. Selectivity of BA-PGMA for Ag(I)

**Figure 7:**
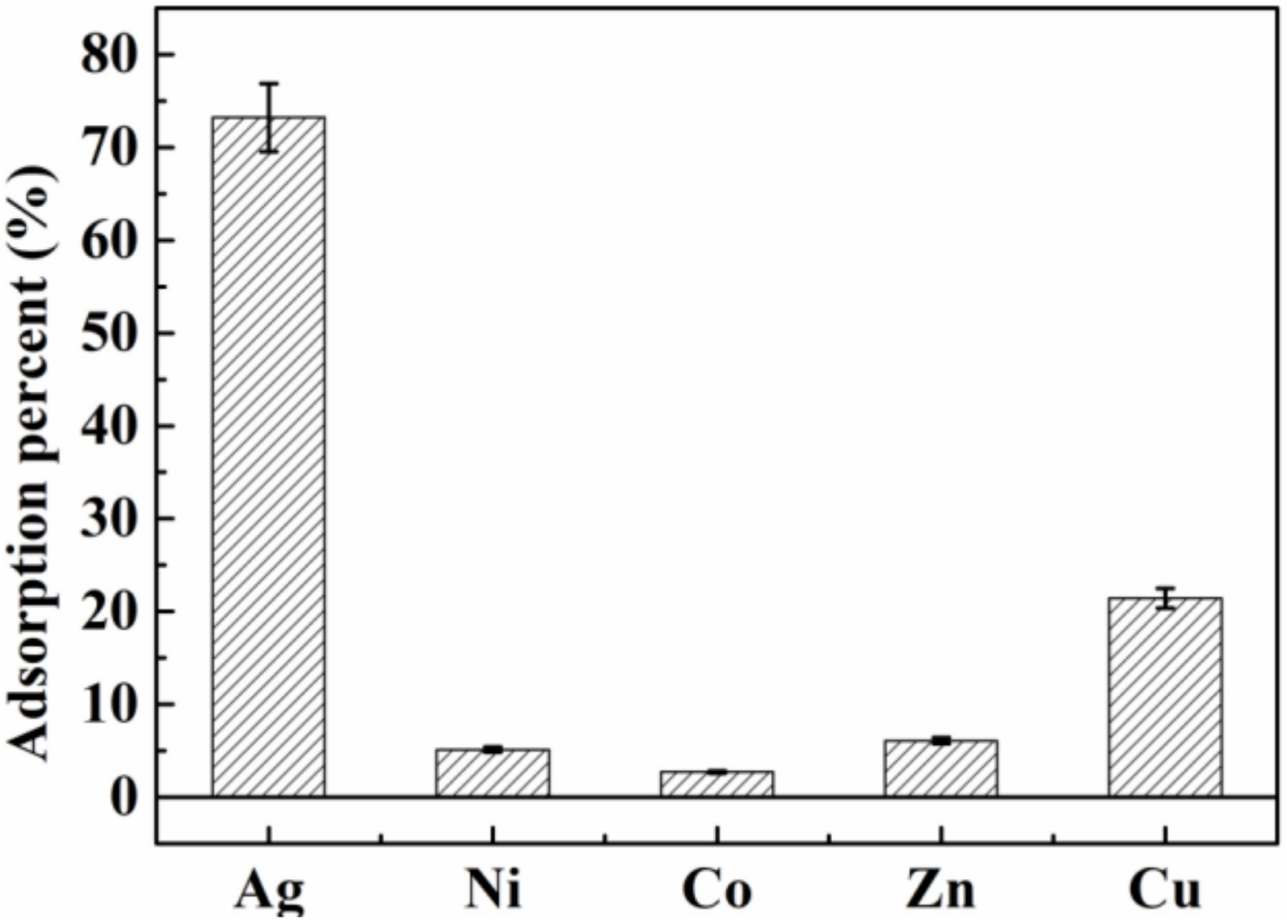
Effect of coexisting ion on the adsorption of Ag(I) by BA-PGMA.

The effect of impurity ions on the adsorption of silver ions was studied. An amount of 10 mg of BA-PGMA was added to 15 mL of the mixed ionic solutions (Ni(II), Cu(II), Ni(II), Zn(II) and Ag(I)) at pH 4. The concentration of each ion was 100 mg/L. The mixed solution was shaken at room temperature for 12 h. It can be seen that the adsorption capacity of other ions is small except for copper ions (Figure 7). Table 6 lists the selectivity parameters for the different ions. The distribution factor (*K*_f_) and the selectivity coefficient (*K*) can be calculated by Equations (13) and (14). By comparison, the adsorption of Ag(I) on BA-PGMA showed a high distribution factor and selectivity coefficient. This further demonstrates that BA-PGMA has good selectivity for Ag(I) adsorption.

(13) $$K_{f}=\frac{U}{C_{\beta }}=\frac{C_{\alpha }-C_{\beta }}{C_{\beta }}\times \frac{V}{m}\times 1000$$

(14) $$K=\frac{K_{f\left({\mathrm{Ag}}^{+}\right)}}{K_{f\left(\mathrm{coexisting}-\mathrm{ions}\right)}}$$

#### 3.2.5. Reuse Performance of BA-PGMA

**Figure 8:**
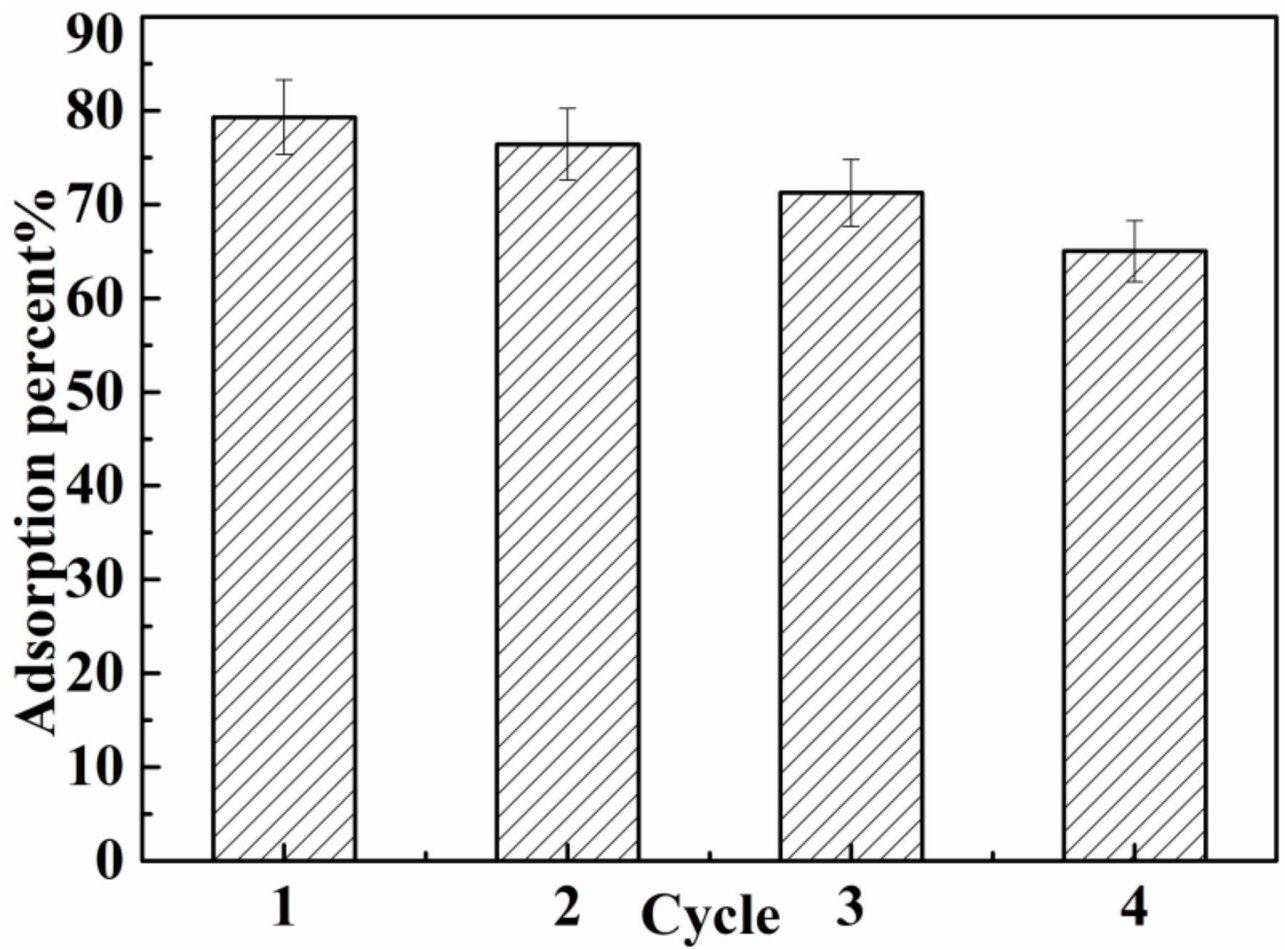
Reusability of BA-PGMA adsorbents.

To test the reusability, 50 mg of BA-PGMA was added to a 75 mL Ag(I) solution with a concentration of 100 mg/L at pH 4, and the mixed solution was shaken for 12 h. After centrifugation, the residue was eluted with a desorbent for 18 h and washed five times with distilled water. The desorbent consisted of nitric acid (3 mol/L) and thiourea (1.5 mol/L). After the experiment, four replicate experiments were performed. Figure 8 shows the reuse effect of BA-PGMA. After four repeated experiments, the removal rate of Ag(I) decreased slightly from 79.29% to 65.01%. This proves that BA-PGMA has good reusability and can be effectively used for silver recovery for at least four cycles.

**Figure 9:**
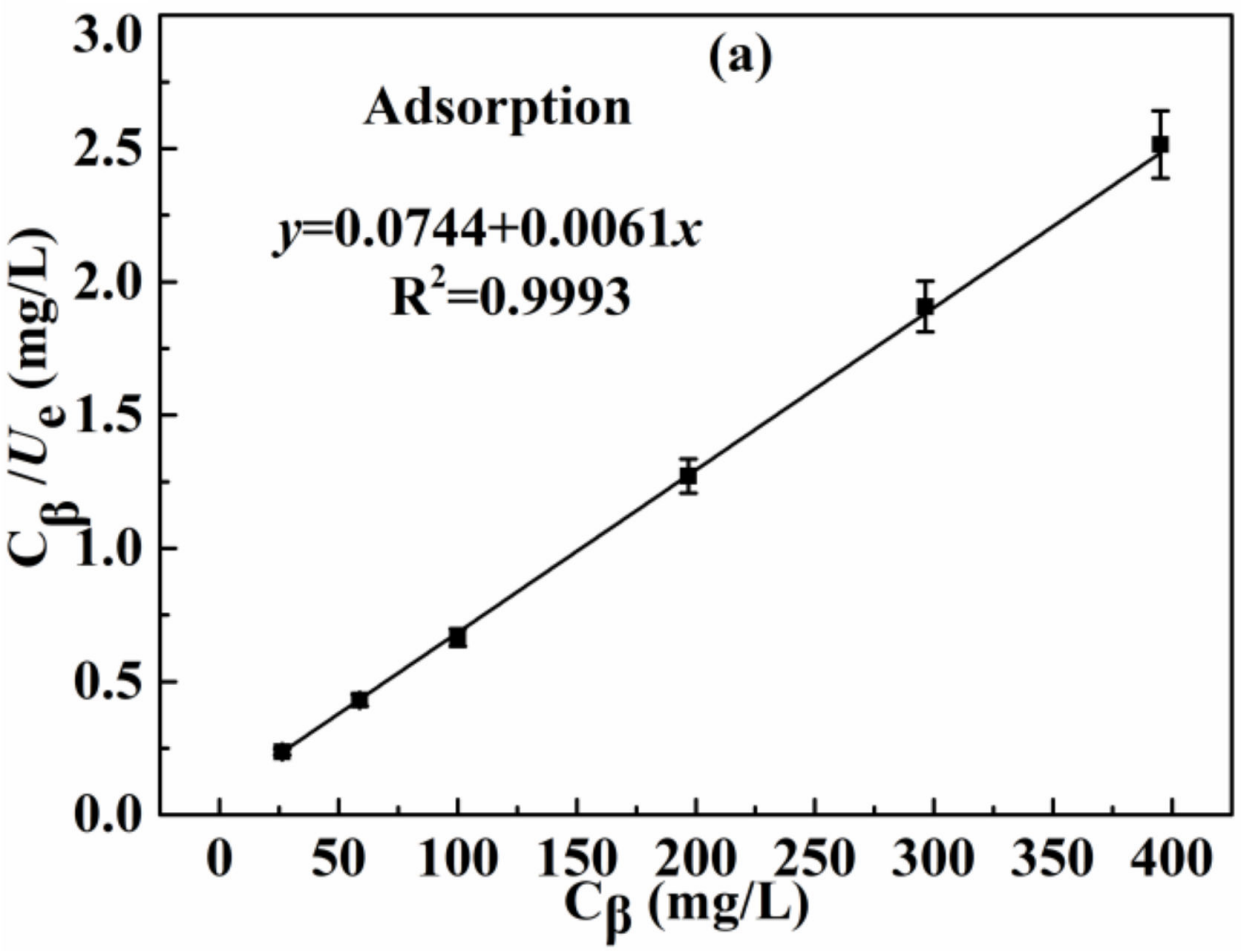

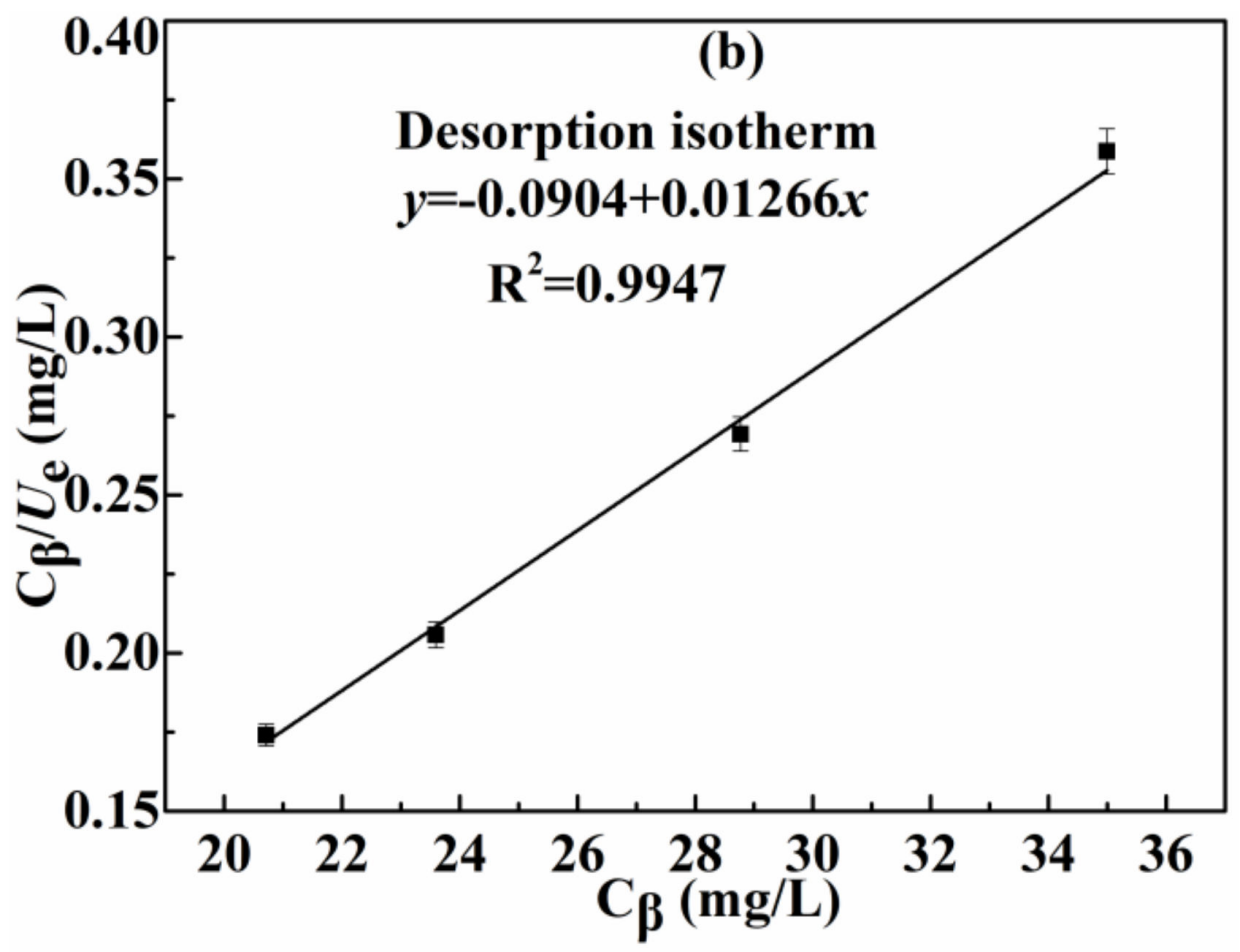
Linear Langmuir plot of adsorption (**a**) and desorption of BA-PGMA (**b**).

The desorption isotherms were used further to investigate the desorption process and the reaction mechanism. Since the adsorption process conforms to the Langmuir model, the model was also used to deal with the desorption process, Equation (15) [56]. *C*_β_, *U*_e_, and *U*_m_ represent the residual concentration of silver ions after adsorption, the adsorption capacity, and single-layer adsorption capacity of silver ions after equilibrium, respectively. *K*_o_ was a constant. The fitting results are showed in Figure 9. By comparison, it can be found that the R values of adsorption (0.9993) and desorption (0.9947) were very close, demonstrating that Ag(I) and the desorbent competed for the active sites on BA-PGMA at the same time [57]. In addition, because the desorbent is charged, it will electrostatically interact with Ag(I), reducing the interaction between BA-PGMA and Ag(I) [58]. It was concluded that the adsorbed Ag(I) was successfully desorbed by desorbent.

(15) $$\frac{C_{\beta }}{U_{e}}=\frac{1}{U_{m\cdot }K_{o}}+\frac{C_{\beta }}{U_{m}}$$

#### 3.2.6. Adsorption Mechanism of Ag(I) by BA-PGMA

**Figure 10:**
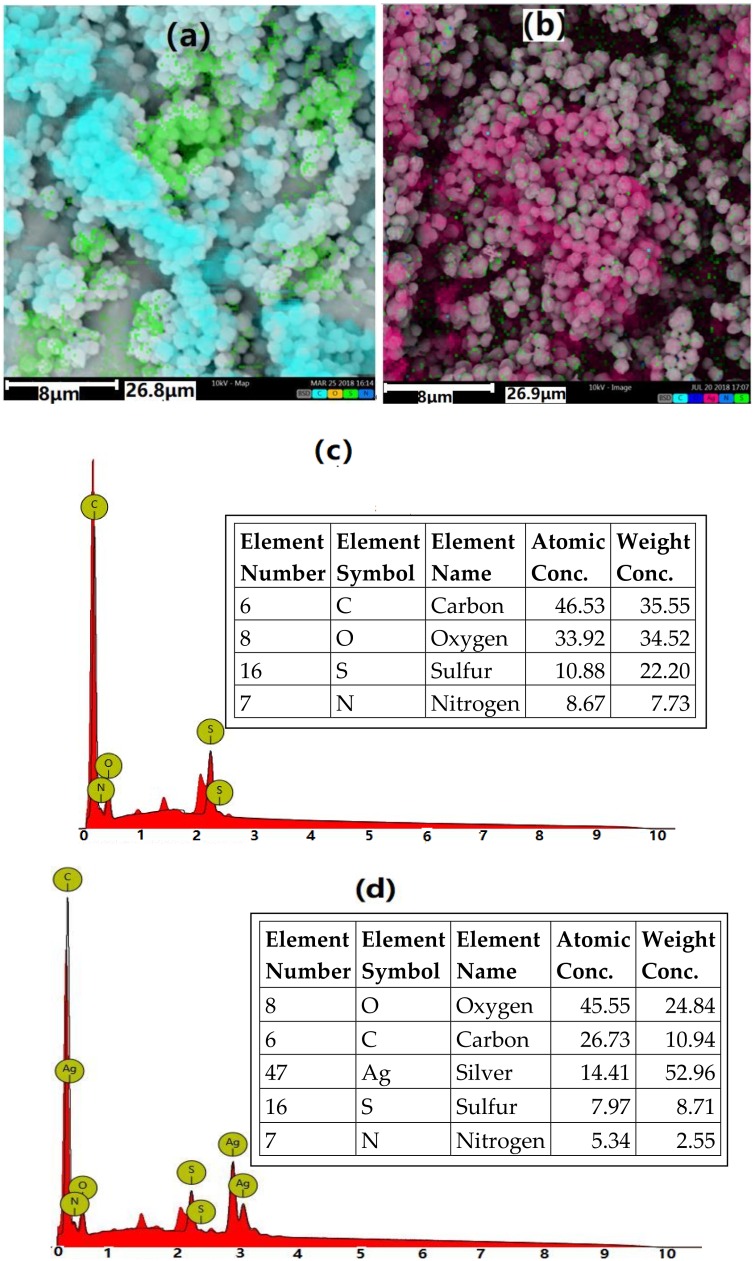
SEM image of BA-PGMA (**a**) and BA-PGMA-Ag (**b**), EDS spectrums of BA-PAMA (**c**) and BA-PGMA-Ag (**d**).

In order to further study the adsorption mechanism of BA-PGMA for Ag(I), BA-PGMA and BA-PGMA-Ag (Ag(I) loaded BA-PGMA defined as BA-PGMA-Ag) were analyzed by SEM and EDS. The results are shown in Figure 10. As can be seen from Figure 10a,b, the BA-PGMA and BA-PGMA-Ag diameters were in the range of 0.1 to 1 µm. The BA-PGMA and BA-PGMA-Ag were scanned by EDS. The scanning energy spectrum is shown in Figure 10c,d. The silver content on BA-PGMA-Ag is very high. Therefore, the combination of Ag(I) with N, O, S was very good, indicating that N, O. and S played an important role in the adsorption process.

**Scheme 2:**
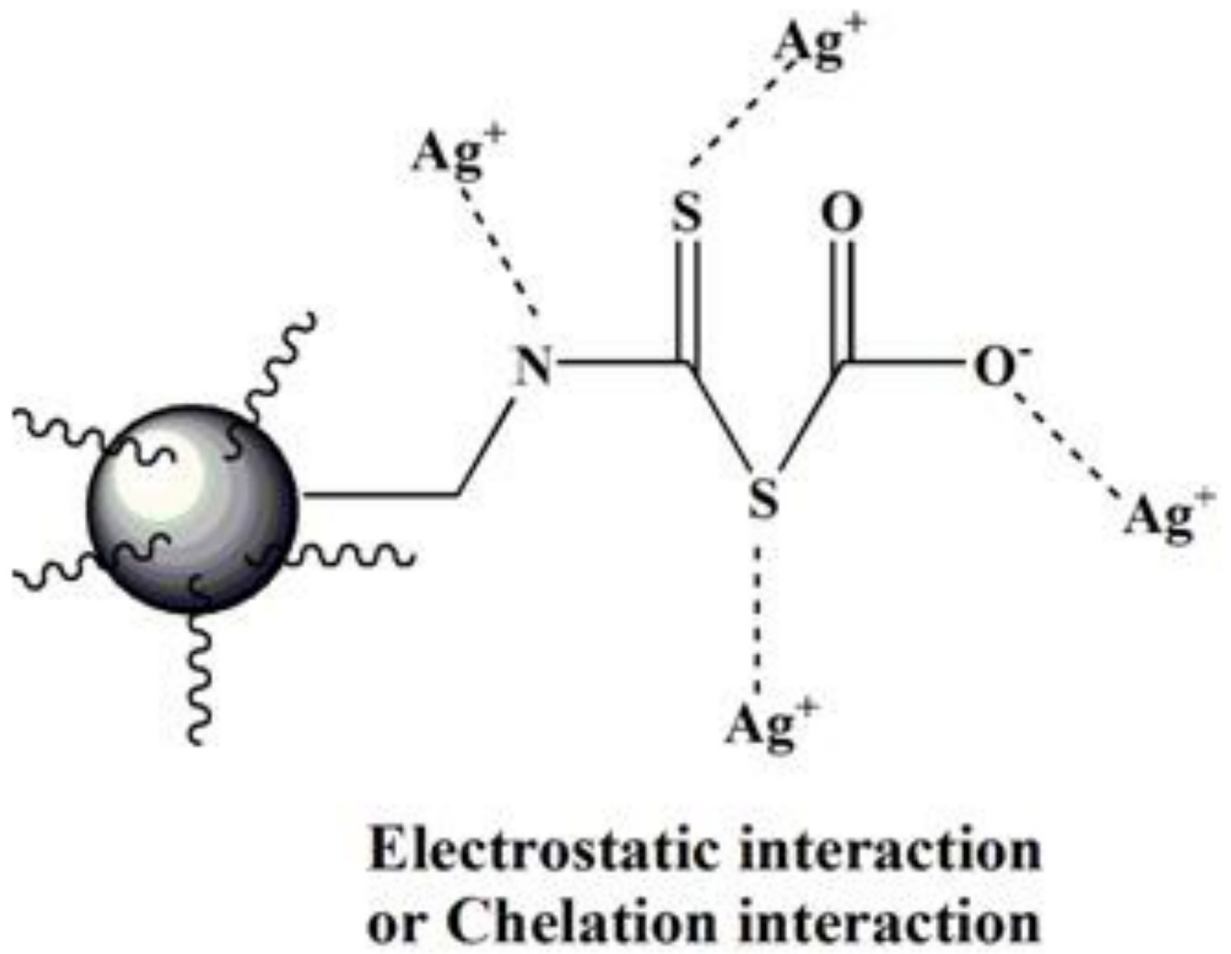
Mechanism of the adsorption of Ag(I) onto BA-PGMA.

**Figure 11:**
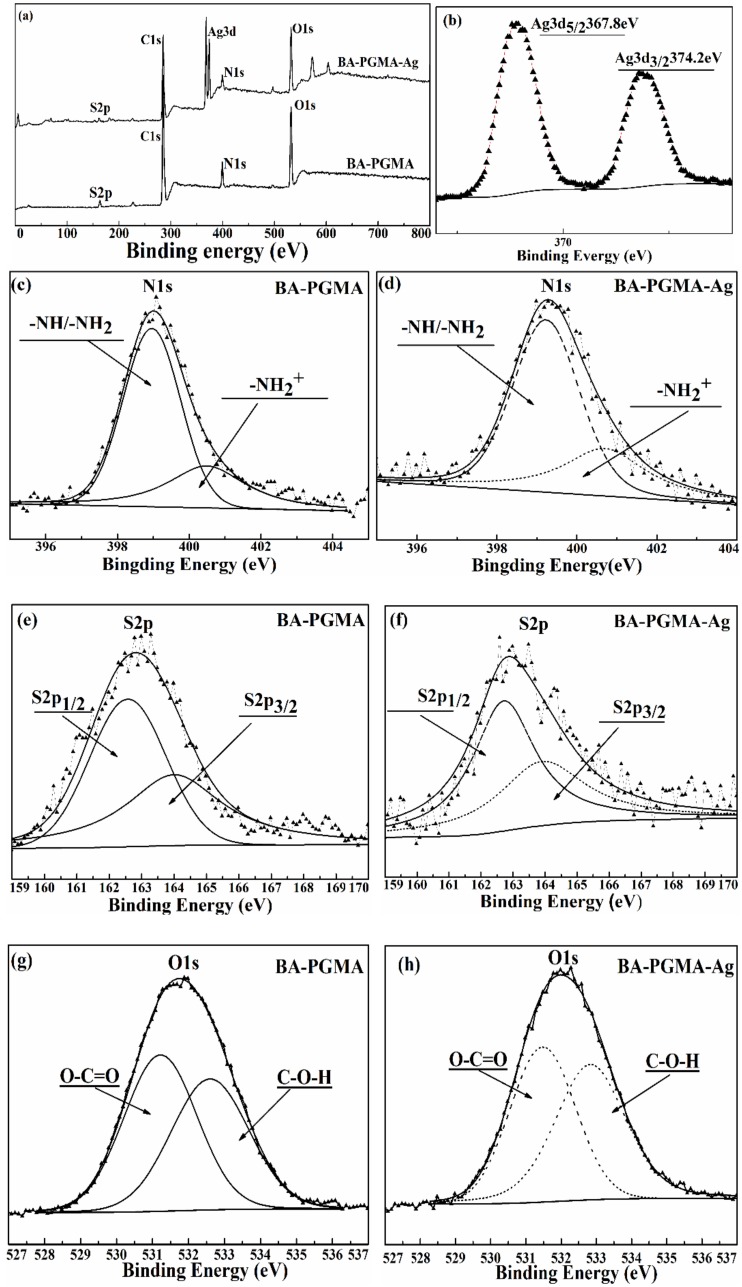
XPS survey scan spectra of BA-PGMA (**a**) and spectra of BA-PGMA-Ag (**b**), N1s spectra of BA-PGMA (**c**) and BA-PGMA-Ag (**d**), S2p spectra BA-PGMA (**e**) and BA-PGMA-Ag (**f**), O1s spectra BA-PGMA (**g**), and BA-PGMA-Ag (**h**).

The XPS analysis of BA-PGMA was carried out before and after the adsorption of Ag(I). Figure 11a is the total spectrum before and after adsorption. The binding energies at 367.8 eV and 374.2 eV were assigned to Ag3d in BA-PGMA-Ag, which were much lower than that of AgNO_3_ [59]. This shows that the electron density was higher than that of free Ag(I). So, Ag(I) participates in the coordination reaction. The binding energy of the N1s group of BA-PGMA and BA-PGMA-Ag is shown in Figure 11c,d. The bonds at 399.1 eV and 400.69 eV represent -NH and HN_2_^+^ in BA-PGMA, respectively. The binding energy of NH_2_^+^ in BA-PGMA-Ag was located at 400.92 eV because Ag(I) covalently bonded with N through lone-pairs electrons. The S2p spectra of BA-PGMA and BA-PGMA-Ag are shown in Figure 11e,f. The binding energy of S_1/2_ and S_3/2_ was shifted from 162.57 eV and 164.1 eV, to 162.69 eV and 163.8 eV, because chelation with Ag(I) and silver thiolate was produced. In Figure 11g,h, the peaks at 531.09 eV and 532.09 eV correspond to the binding energies of O-C=O and C-O-H, respectively. Due to the coordination with Ag(I), the potential at 532.09 eV for C-O-H in BA-PGMA shifted to 532.82 eV in BA-PGMA-Ag [60]. The main mechanism is electrostatic interaction and chelation (Scheme 2).

#### 3.2.7. Thermodynamic Studies

**Figure 12:**
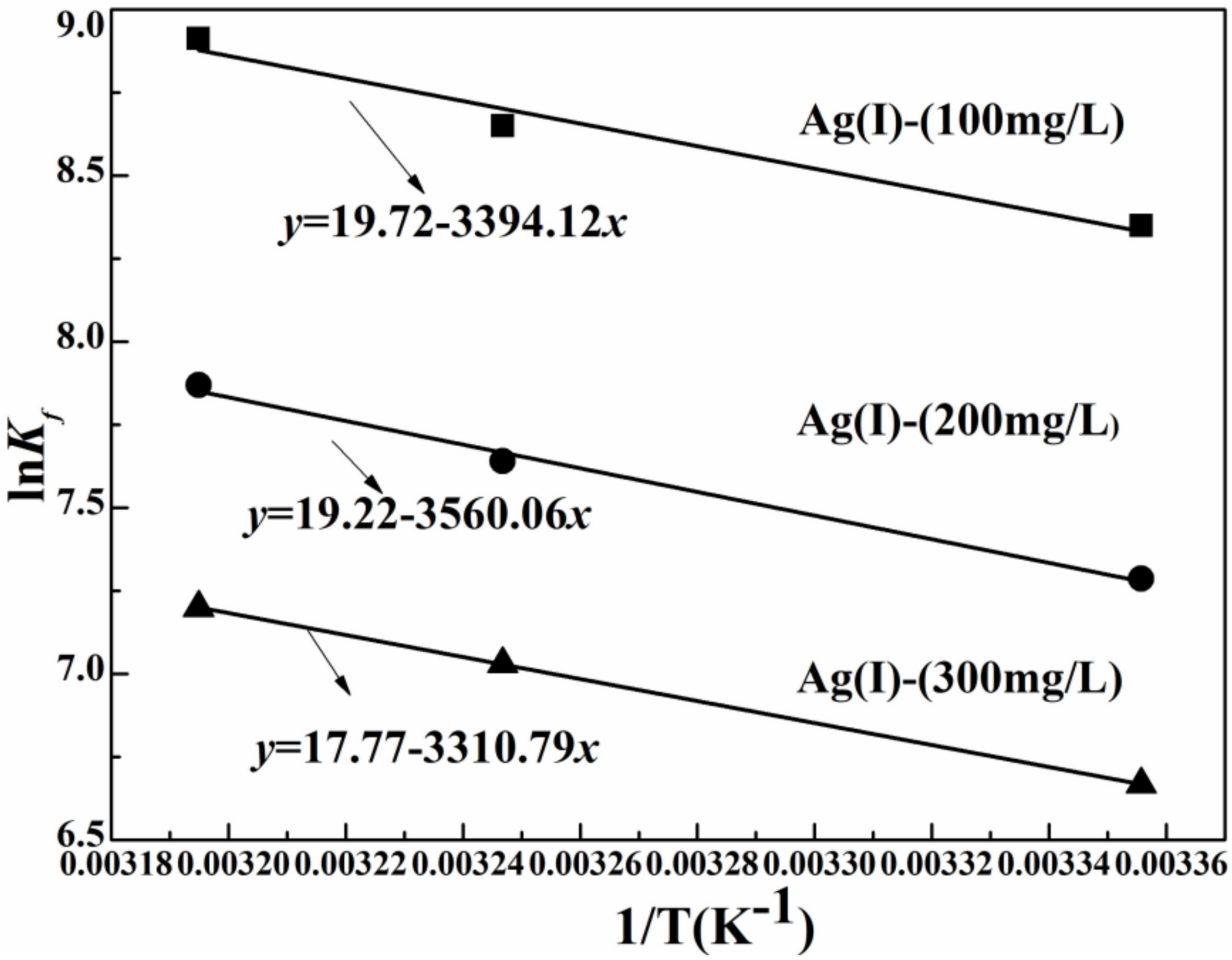
Effect of temperature on the Ag(I) adsorption capacity.

In order to study the adsorption thermodynamics of Ag(I) on BA-PGMA, the effect of temperature on the adsorption of BA-PGMA was studied when the concentration of Ag(I) was constant. An amount of 10 mg of BA-PGMA was added to a 15 mL Ag(I) solution and shaken at 250 rpm for 300 min at 298 K, 308 K, and 318 K, respectively. The changes of Gibbs free energy (Δ*G*^θ^), standard entropy (Δ*S*^θ^), and standard entropy (Δ*H*^θ^) were studied. The relationship between them can be expressed by the following formula [61]. Three Ag(I) concentrations (100 mg/L, 200 mg/L and 300 mg/L) were undertaken.

(16) $$\Delta G^{\theta }=-RT\ln K_{f}=\Delta H^{\theta }-T\cdot \Delta S^{\theta }$$

(17) $$\ln K_{f}=\frac{-\Delta G^{\theta }}{RT}=\frac{\Delta S^{\theta }}{R}-\frac{\Delta H^{\theta }}{RT}$$

where, *k*_f_ represent the distribution coefficient and can be calculated by Equation (13), *R* is the gas constant (8.314 J/mol/K), and *T* is the temperature in Kelvin (K). According to Equation (16), the data obtained by experiment was linearly fitted as ln(*K*_f_) versus 1/T (*K*^−1^) and the fitting result is shown in Figure 12.

The standard entropy (Δ*S*^θ^) and the standard entropy (Δ*H*^θ^) were calculated by the intercept and slope of Figure 12. [62] It can also be seen that the partition coefficient (*K*_f_) decreased with the reciprocal of temperature. When the concentration was constant, the higher the temperature, the better is the adsorption effect. The relevant parameters are listed in Table 7. These results indicate that the adsorption process was spontaneous and endothermic in nature, with irregular increase of randomness in the reaction interface of the BA-PGMA/Ag(I) [63].

## 4. Conclusions

In this work, a new adsorbent (BA-PGMA) was synthesized by modifying poly(glycidyl methacrylate) to selectively absorb Ag(I) from an aqueous solution. The adsorbent was characterized by FI-TR, SEM, and XPS. The batch experiments showed the optimum pH was 4.0. The maximum absorption capacity of Ag(I) at pH 4 and room temperature was 155.05 mg/g. The adsorption equilibrium time was 300 min. The adsorption isotherms are consistent with Langmuir and D-R models. The adsorption kinetics is fitted with a pseudo-secondary model. The BA-PGMA has good selectivity for Ag(I) for coexisting ions (Cu^2+^, Ni^2+^, Co^2+^, Zn^2+^). At the same time, the adsorption mechanism is of a chelation and electrostatic action. Finally, the adsorbent could be reused to remove silver ions. The adsorption process is exothermic, feasible, and spontaneous. Therefore, it can be concluded that BA-PGMA is a promising adsorbent for recovering Ag(I) from wastewater.

## Figures and Tables

**Table 1:** Kinetic parameters for Ag(I) adsorption by BA-PGMA.

**First-Order Model**	*K*_a_ (g/(mg·min))	*U*_e_ (mg/g)	*R*^2^ Reduced Chi-Square
	0.06	108.51	0.9124 12.75
**Second-Order Model**	*K*_b_ (g/(mg·min))	*U*_e_ (mg/g)	*R*^2^ Reduced Chi-Square
	8.20	116.84	0.9914 1.24
**Intra-Particle Diffusion Model**	*K_c_* (mg/(g·min^1/2^)		*R* ^2^
	3.32		0.7993

**Table 2:** The parameters of the three stages of the I-R model.

**Stage**	***K_c_***	***R*** **^2^**	***C***
I	11.46	0.9953	22.95
II	2.16	0.8583	78.62
III	0.03	−0.8881	114.61

**Table 3:** Adsorption isotherms parameters for Ag(I) on BA-PGMA.

**Freundlich**	*K*_h_	*n*	*R*^2^ Reduced Chi-Sqr
	79.02	8.19	0.8387 8.39
**Langmuir**	*U*_m_ (mg/g)	*K_i_*	*R*^2^ Reduced Chi-Sqr
	163.46	0.08	0.9927 0.37
**Temkin**	*K*_j_	*V_T_*	*R*^2^ Reduced Chi-Sqr
	29.22	17.49	0.8747 6.52
**Hill**	*K*_L_	*N*	*R*^2^ Reduced Chi-Sqr
	12.14	0.91	0.9848 0.78
**D-R**	ω	*E* (kj/mol)	*R*^2^ Reduced Chi-Sqr
	0.0041	11.04	0.9974 0.13

**Table 4:** The Langmuir-based *R*_L_.

*C*_α_ (mg/L)	*R*_L_
100	0.0808
150	0.0554
200	0.0421
300	0.0285
400	0.0215
500	0.0172

**Table 5:** Comparison of the adsorption amount of Ag(I) and adsorbent reported in the literature.

Sorbent	*U*_m_ (mg/g)	Reference
Natural Clinoptilolites	31.44	[48]
Active carbon	32.80	[49]
Nanocelluloses and their phosphorylated derivatives	56	[50]
MFT chelating resin	60.05	[51]
Bentonite clay	61.48	[52]
Ion-imprinted chitosan gel beads	80.50	[53]
Ag^+^-imprinted chitosan gel bead	89.20	[54]
Valonia tannin resin	97.08	[55]
BA-PGMA	157.05	This work

**Table 6:** The values of *K*_f_ and *K* of coexisting ions.

Coexisting Ions	*K*_f_ (mL/g)	*K*
Ag^+^	4097.0	-
Ni^2+^	81.3	50.4
Co^2+^	41.9	97.8
Zn^2+^	97.4	42.1
Cu^2+^	409.1	10.0

**Table 7:** Thermodynamic parameters of Ag(I) adsorption onto BA-PGMA.

Initial Ag(I) Concentration	*T* (K)	Δ*S*^θ^ (J∙K^−1^∙moL^−1^)	ΔH^θ^ (kJ∙moL^−1^)	Δ*G*^θ^ (kJ∙moL^−1^)
100 (mg/L)	25 °C (298 K)	163.95	28.22	−20.68
	35 °C (303 K)			−21.79
	45 °C (313 K)		-	−23.19
200 (mg/L)	25 °C (298 K)	159.7	29.60	−18.05
	35 °C (303 K)			−19.25
	45 °C (313 K)		-	−20.48
300 (mg/L)	25 °C (298 K)	147.73	27.52	−16.50
	35 °C (303 K)			−17.71
	45 °C (313 K)		-	−18.74
